# RSV-Induced H3K4 Demethylase KDM5B Leads to Regulation of Dendritic Cell-Derived Innate Cytokines and Exacerbates Pathogenesis *In Vivo*

**DOI:** 10.1371/journal.ppat.1004978

**Published:** 2015-06-17

**Authors:** Catherine Ptaschinski, Sumanta Mukherjee, Martin L. Moore, Mareike Albert, Kristian Helin, Steven L. Kunkel, Nicholas W. Lukacs

**Affiliations:** 1 Department of Pathology, University of Michigan, Ann Arbor, Michigan, United States of America; 2 Department of Pediatrics, Emory University, and Children’s Healthcare of Atlanta, Atlanta, Georgia, United States of America; 3 Biotech Research and Innovation Centre, Centre for Epigenetics, and Danish Stem Cell Center, University of Copenhagen, Copenhagen, Denmark; University of North Carolina at Chapel Hill, UNITED STATES

## Abstract

Respiratory syncytial virus (RSV) infection can result in severe disease partially due to its ability to interfere with the initiation of Th1 responses targeting the production of type I interferons (IFN) and promoting a Th2 immune environment. Epigenetic modulation of gene transcription has been shown to be important in regulating inflammatory pathways. RSV-infected bone marrow-derived DCs (BMDCs) upregulated expression of *Kdm5b/Jarid1b* H3K4 demethylase. *Kdm5b*-specific siRNA inhibition in BMDC led to a 10-fold increase in IFN-β as well as increases in IL-6 and TNF-α compared to control-transfected cells. The generation of *Kdm5b* ^fl/fl^-CD11c-Cre^+^ mice recapitulated the latter results during *in vitro* DC activation showing innate cytokine modulation. *In vivo*, infection of *Kdm5b* ^fl/fl^-CD11c-Cre^+^ mice with RSV resulted in higher production of IFN-γ and reduced IL-4 and IL-5 compared to littermate controls, with significantly decreased inflammation, IL-13, and mucus production in the lungs. Sensitization with RSV-infected DCs into the airways of naïve mice led to an exacerbated response when mice were challenged with live RSV infection. When *Kdm5b* was blocked in DCs with siRNA or DCs from *Kdm5b* ^fl/fl^-CD11c-CRE mice were used, the exacerbated response was abrogated. Importantly, human monocyte-derived DCs treated with a chemical inhibitor for KDM5B resulted in increased innate cytokine levels as well as elicited decreased Th2 cytokines when co-cultured with RSV reactivated CD4^+^ T cells. These results suggest that KDM5B acts to repress type I IFN and other innate cytokines to promote an altered immune response following RSV infection that contributes to development of chronic disease.

## Author Summary

Respiratory syncytial virus (RSV) is a significant public health concern. Nearly all children are infected by two years of age, and severe infection often results in hospitalization. There is no vaccine for RSV, and previous attempts have resulted in increased disease severity in immunized children once they were exposed to the virus. Therefore, a better understanding of how RSV directs the immune response is needed. In this study, we found that the protein KDM5B regulates an epigenetic mechanism that directs the immune response to RSV. KDM5B suppressed the activation of key antiviral signals in dendritic cells, and inhibition of KDM5B led to gene activation and increased antiviral function. This correlated with decreased pathology in the lungs. Therefore, our data suggest that new attempts at designing a vaccine should consider the effects of vaccination on dendritic cells, and should consider strategies that will increase antiviral signals from dendritic cells.

## Introduction

Respiratory syncytial virus (RSV) is a significant burden to healthcare worldwide. In the United States nearly all children have been infected by age two [1,2]. Severe infections are the leading cause of bronchiolitis in children, resulting in up to 125,000 hospitalizations in the US each year [3,4]. Furthermore, infants who are hospitalized with severe disease are 3–4 times more likely to develop asthma later in life [5,6], as RSV interferes with the initiation of the adaptive immune response leading to an altered immune environment in the lungs. One consequence is the recruitment of T cells that produce interleukin (IL)-4, IL-5 and IL-13, which are cytokines involved in the pathogenesis of lung disease, including asthma. Since RSV infection does not result in a strong memory response, repeated infections are common throughout childhood and for adults [7]. Currently, there is no vaccine for RSV. Attempts to create a formalin-inactivated vaccine resulted in more severe infection upon exposure to the virus in those children who were vaccinated compared to unvaccinated children [8]. Thus, the need to understand the immune response to RSV at the molecular level is critical to develop better therapeutics and aid in the development of effective vaccines.

Dendritic cells (DCs) residing in the airway are among the first cells to encounter an RSV infection. An important role for DCs is the production of IL-12, which is critical to initiate a Th1 response following infection with a virus. Viral infection also results in the recognition of pathogen-associated molecular patterns (PAMPs) such as viral nucleic acids, including double-stranded RNA. DCs express toll-like receptors (TLRs) that recognize these PAMPs. Viral RNA recognition signals induce the production of type I IFNs, including IFN-β, thereby leading to an antiviral state in nearby cells and initiating the proper T cell response. However, while other respiratory viruses such as influenza drive a strong IFN-β response, RSV infection suppresses this response, which is partially due to viral non-structural protein interactions with activation factors, resulting in the suppression of both helicase RIG-I [9] and the transcription factor NF-γB [10]. These events not only allow for viral replication, but also promote an altered immune environment in the lungs, characterized by mucus hypersecretion [11]. As DCs are important cells in directing the T cell response, it is imperative to understand the mechanisms by which RSV interferes with proper DC function.

Recent studies have documented that epigenetic modifications of immune cells at the chromatin level contribute to either activation or repression of specific genes. This regulation can occur by several mechanisms, including methylation, acetylation and phosphorylation of histone tails. Histone methylation can occur on lysine and arginine residues, and contribute to activation or inhibition of transcription. For example, the addition of methyl groups on lysine (K) 27 of histone (H) 3 (i.e. H3K27) is associated with repression of gene transcription, whereas H3K4 methylation correlates with active gene transcription [12–14]. This process is controlled by methyltransferases that add methyl groups and demethylases that remove them. There is accumulating evidence that epigenetic modulation is an important component of immune cell phenotype and function. For example, the cytokines typically produced by Th2 cells, including IL-4, IL-5 and IL-13, are silenced in Th1 cells through histone modification [15]. In addition, Th17 cells have increased H3K4 methylation, a gene activating mark, at the Th17 promoter [16]. In DCs, IL-12 production is decreased following severe sepsis due to decreased H3K4 methylation altering subsequent immune responses [17]. Despite these inroads, data is lacking regarding the contribution of epigenetic regulation to immune cell activation. In the present studies an upregulation of *Kdm5b*, coding for an H3K4 demethylase, following RSV infection of DCs was observed. As H3K4 methylation is an activating mark, this demethylase has the potential to repress transcription. A role for KDM5B in transcriptional repression has been reported in cancer cells including melanoma, breast cancer and prostate cancer [18–20]. Here we show that KDM5B has a critical role in preventing the activation of DCs during RSV-induced immune responses. Our results show that decreasing *Kdm5b* expression by siRNA, chemical inhibition or genetic deletion prior to RSV infection leads to an increase in the production of IFN-β and other inflammatory cytokines compared to uninfected controls, as well as decreased Th2 pathogenesis *in vivo* thus linking *Kdm5b* expression with disease exacerbation during RSV infection.

## Results

### Expression of histone lysine demethylases and Kdm5b following infection of BMDCs with RSV

**Fig 1:**
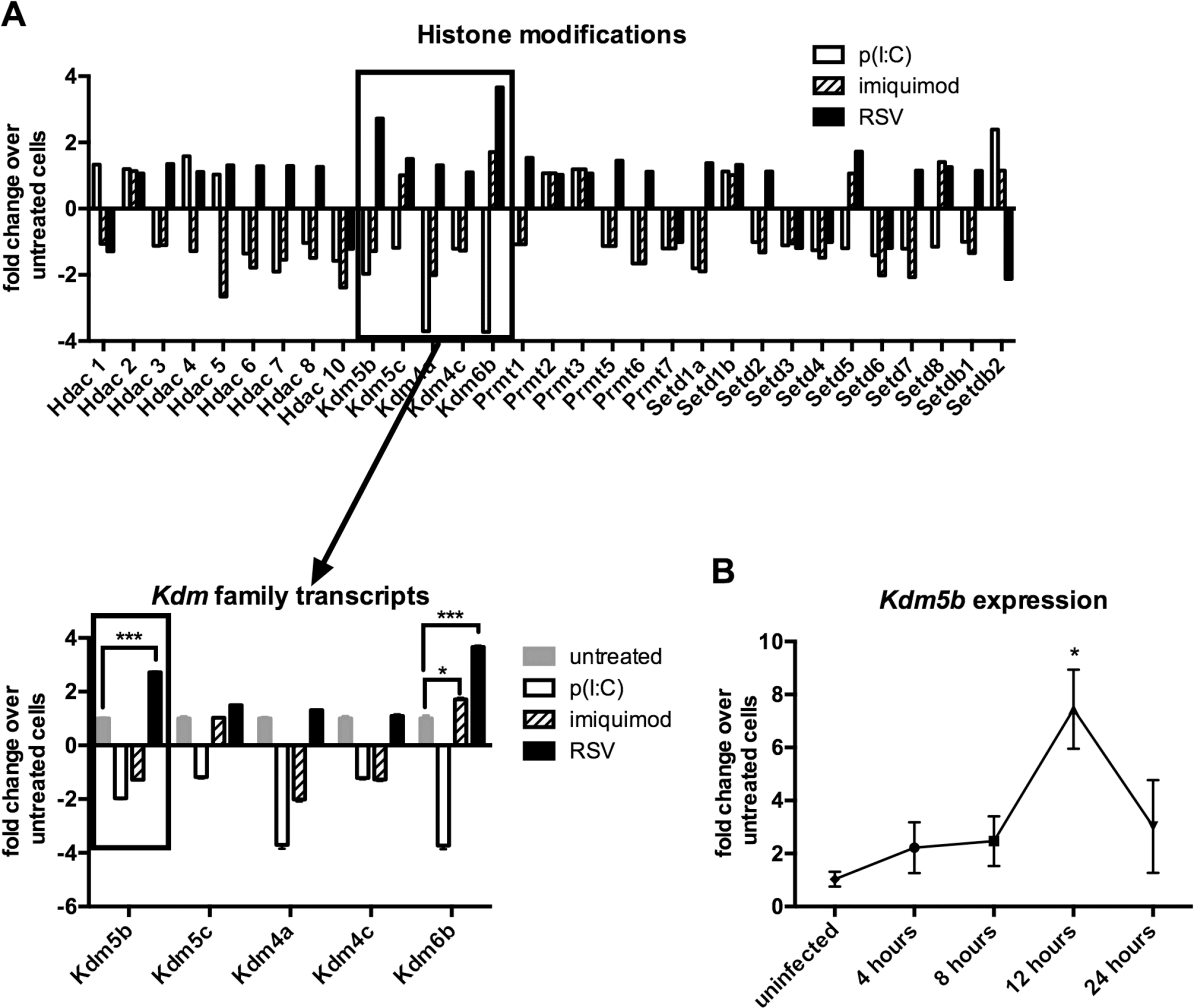
*Kdm5b* expression increases following infection of BMDCs with RSV. Bone marrow-derived dendritic cells were infected with RSV (MOI = 1) or treated with poly(I:C) (20 μg/ml) or imiquimod (1 μg/ml) for four hours. RNA was extracted and reverse transcribed, and the cDNA was used in a PCR array to measure changes in the expression of epigenetic enzymes (A), including the histone lysine demethylase family. Expression of *Kdm5b* was measured over a time course following infection with RSV (B). n = 3–5 samples/group, and data are representative of three independent experiments. *p<0.05.

A previous report has identified a role for epigenetic regulation in immune cells following viral infection [21]. As DCs are critical for priming the T cell response to RSV infection, studies were initiated to determine whether exposing DCs to RSV resulted in changes in the expression of epigenetic factors in the DCs. BMDCs were infected with RSV or activated by p(I:C) or imiquimod, the ligands for TLR3 and TLR7 respectively, as RSV is known to activate cells through both TLR3 and TLR7 [22,23], in addition to other mechanisms. In order to observe early gene expression of epigenetic enzymes, RNA was harvested at 4 hours post treatment to examine transcription levels of genes coding for “epigenetic” enzymes by qPCR array. Several classes of enzymes were analyzed including histone deacetylases (HDACs), histone lysine demethylases (KDMs), protein arginine methyltransferases (PRMTs), and histone lysine methyltransferases (KMTs) (Fig 1A). A defining observation was the upregulation of *Kdm5b* demethylase by RSV in contrast to the downregulation of this enzyme by stimulation through TLR3 and TLR7 (Fig 1A). While *Kdm6b* was upregulated by RSV infection of DCs, this enzyme was also significantly upregulated by treatment of cells with imiquimod. Because *Kdm5b* was upregulated only by RSV, studies focused on *Kdm5b* as a potential unique enzyme in the DC response to RSV. PCR analysis confirmed the peak expression of *Kdm5b* in BMDCs at 12 hours following RSV infection (Fig 1B). Furthermore, while *Kdm5b* was upregulated in BMDCs infected with RSV, it was not upregulated by influenza (H1N1) virus, nor in RSV-infected epithelial cells or alveolar macrophages (S1 Fig). Therefore, studies focused on H3K4 demethylase *Kdm5b* and its role on perturbing critical innate immune genes in DCs.

### Increased expression of pro-inflammatory cytokines following disruption of KDM5B function

**Fig 2:**
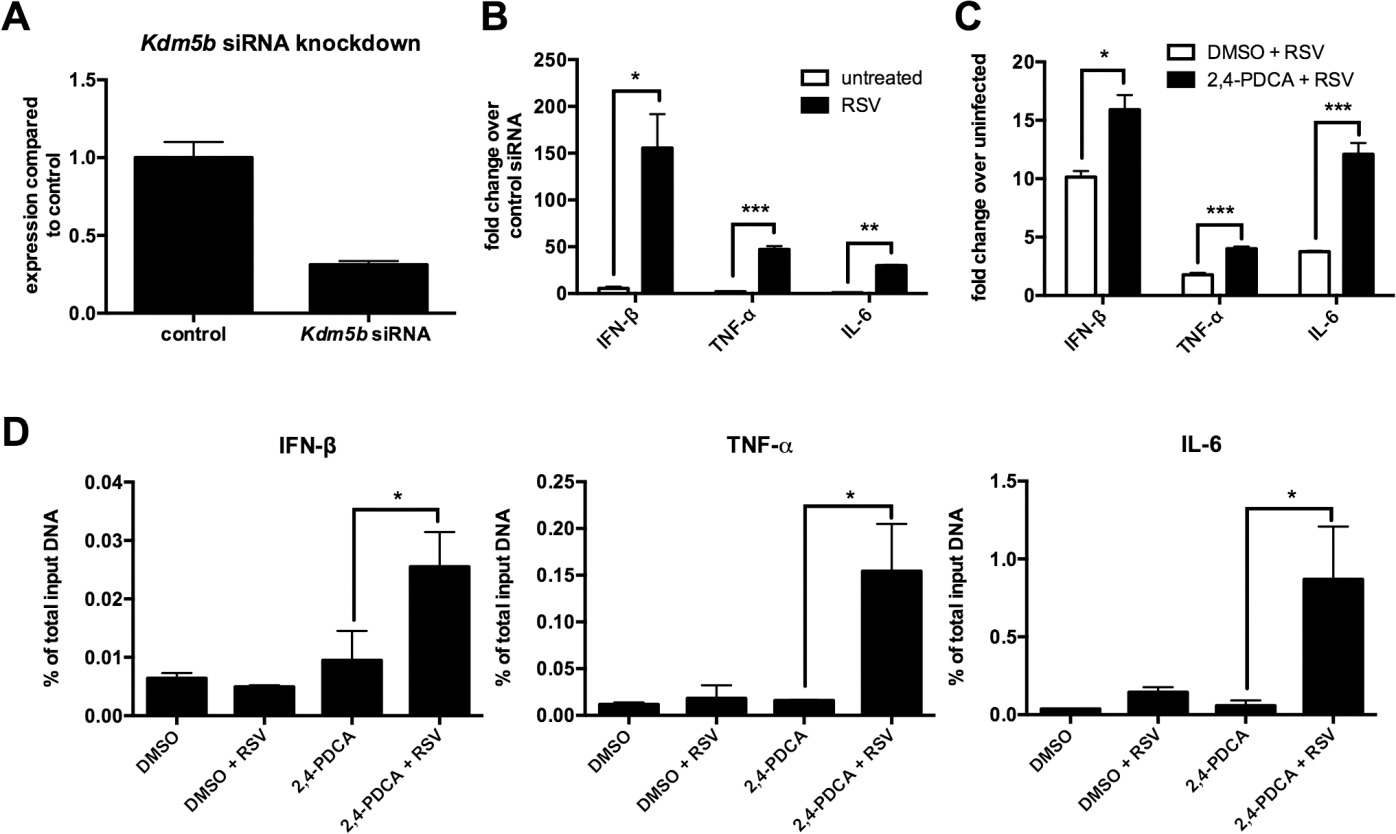
siRNA knockdown of *Kdm5b* leads to increased cytokine and chemokine gene expression. (A) BMDCs were transfected with *Kdm5b*-specific siRNA or non-targeting control siRNA (1 μM). Total RNA was extracted from the cells and gene expression levels measured by qPCR. n = 6 from two combined experiments. (B) Transcript levels of *Ifnb*, *Tnf* and *Il6* were measured from *Kdm5b*-siRNA transfected BMDCs and were compared to scrambled siRNA control following infection with RSV. n = 3 samples/group and is representative of two independent experiments. (C) BMDCs were treated with 1μM 2,4-PDCA or 0.1% DMSO for 24 hours and subsequently infected with RSV for 24 hours. Transcript levels were measured by qPCR. n = 4 samples/group and is representative of two independent experiments. (D) DMSO or 2,4-PDCA-treated BMDCs were infected with RSV for 24 hours. A ChIP assay was performed to determine the H3K4me3 status at the promoter regions of the *Ifnb*, *Tnf* and *Il6* genes. Data are representative of three different experiments, with each sample run in duplicate. n = 4 samples/group. *p<0.05, **p<0.01, ***p<0.001.

To determine whether KDM5B affects DC function, specific siRNA was used to knock down *Kdm5b* resulting in >70% reduction in expression levels (Fig 2A). Previous reports have indicated that RSV, unlike many viruses, is a poor inducer of type I IFN, including IFN-β [9,10]. BMDCs infected with RSV produced low levels of IFN-β at both 4 and 24 hours, whereas H1N1 virus produced very high levels (S2 Fig). We therefore hypothesized that the increase in KDM5B in BMDCs contributed to the suppression of type I IFN production and that knocking down *Kdm5b* expression would result in increased IFN-β. Following *in vitro* treatment of BMDCs with *Kdm5b*-specific siRNA or with a scrambled siRNA control, significantly increased expression levels of *Ifnb*, as well as the pro-inflammatory cytokines *Tnfa* and *Il6* were observed in *in vitro* RSV-infected cells compared to sham-infected BMDCs (Fig 2B). To determine whether APC function was affected by *Kdm5b* siRNA or inhibitor treatment, MHC-II expression on the cell surface of BMDCs was measured, as well as expression of the co-stimulatory molecules CD80 and CD86. No differences in any maturation markers were noticed in treated cells compared to controls (S3 Fig). Furthermore, when a chemical inhibitor, 2,4-pyridinedicarboxylic acid (2,4-PDCA), was used to block the function of KDM5B [24,25] prior to RSV infection, significantly higher levels of *Ifnb*, *Tnfa* and *Il6* transcripts compared to controls were observed (Fig 2C). While this inhibitor also interacts with other KDM family members, it has the highest specificity for KDM5B. Thus, two independent approaches to block KDM5B function demonstrated an altered immune response resulting in increases of critical innate cytokines.

KDM5B catalyzes the demethylation of H3K4me3 and H3K4me2. As H3K4me3 is associated with active gene transcription, the activity of KDM5B in removing a methyl group leads to decreased promoter activity and decreased gene transcription. Since blocking KDM5B activity led to increased levels of proinflammatory cytokines, we hypothesized that blocking the demethylase activity would lead to greater H3K4me3 at the promoters of these cytokines. To test this hypothesis, a ChIP assay using an anti-H3K4me3 antibody was performed on cells treated with 2,4-PDCA, and primers designed to recognize the promoter regions of *Ifnb*, *Tnf* and *Il6* were used. Treatment of DC with 2,4-PDCA prior to infection with RSV led to an increase of H3K4me3 compared to controls on all three cytokine promoters (Fig 2D). Conversely, it was found that there were no differences in H3K4 methylation on the promoters of *Il10* and *Il12*, which are two cytokines that were unchanged following RSV infection of *Kdm5b*-deficient DCs (S4 Fig). These results indicate that the KDM5B demethylase activity acts on the promoters of specific inflammatory genes, thereby suppressing transcription.

### Inhibition of KDM5B in human DCs results in decreased inflammatory gene activity and lower IL-4 production by co-cultured CD4^+^ T cells

**Fig 3:**
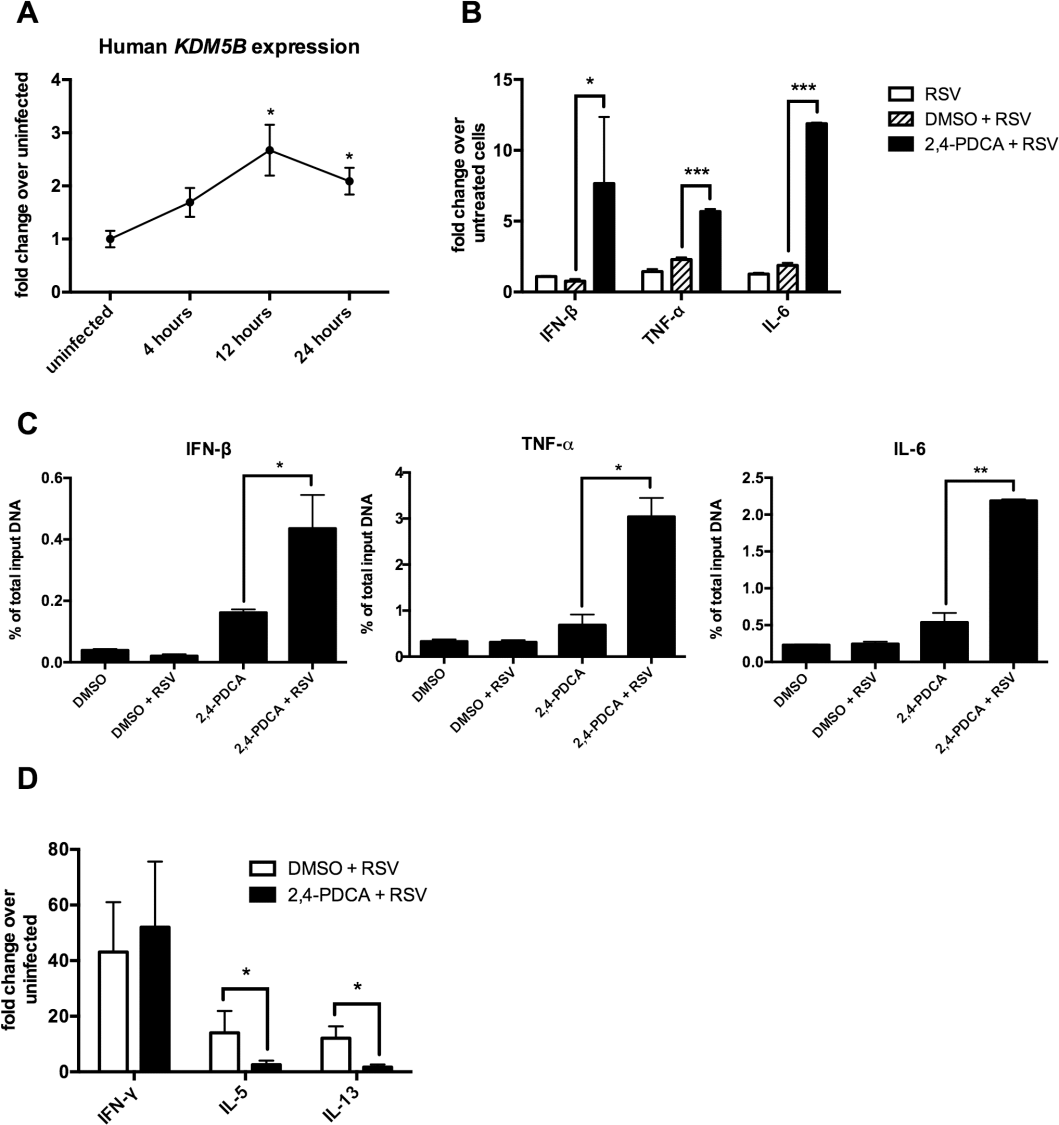
Inhibiting KDM5B in human monocyte-derived DCs leads to increased cytokine production. (A) DCs were grown from blood monocytes and infected with RSV. RNA was isolated and *KDM5B* expression was determined by qPCR. n = 6 samples from three pooled donors. (B) DCs were treated with DMSO or 2,4-PDCA for 24 hours, and were then infected with RSV for 24 hours. Cytokine levels were measured by qPCR. n = 3 samples/group and is representative of three independent experiments. (C) DMSO or 2,4-PDCA-treated BMDCs were infected with RSV for 24 hours. A ChIP assay was performed to determine the H3K4me3 status at the promoter regions of the *IFNB*, *TNF* and *IL6* genes. n = 4 samples/group. (D) DCs were treated with DMSO or 2,4-PDCA and infected with RSV. Autologous CD4^+^ T cells were cultured with the DCs for 48 hours. mRNA was extracted and cytokines levels measured by qPCR. n = 3 samples/group and data are representative of two independent experiments. p<0.05,**p<0.01 ***p<0.001.

The above data show that inhibiting KDM5B function leads to increased innate cytokine production after RSV infection. To determine the role of KDM5B in human cells, human monocyte-derived DCs (MoDCs) cultured from peripheral blood monocytes were used. As shown in Fig 3A, *KDM5B* is significantly upregulated in MoDCs at 12 and 24 hours following RSV infection, and although the degree of upregulation is less pronounced than in mouse DCs, the kinetics are very similar (Fig 3A). To assess the role of KDM5B in human DCs, the function of KDM5B was inhibited by treating the cells with 2,4-PDCA for 24 hours, followed by RSV infection. Similar to observations in mouse cells treated with 2,4-PDCA, inhibiting KDM5B in human MoDCs led to increased production of *IFNB*, *TNF* and *IL6* compared to RSV alone or DMSO control (Fig 3B). To determine whether the presence of the inhibitor affected the H3K4me3 status of these genes, a ChIP analysis for H3K4 was performed and examined the promoter regions of specific innate cytokine genes. Interestingly, MoDCs exhibited a slight increase in promoter methylation following incubation with the inhibitor alone, which was further increased when the cells were infected with RSV (Fig 3C). Similar to observations in mouse DCs, no change in methylation at the *IL10* an *IL12* promoters was observed (S4 Fig). Finally, infected MoDCs that had been treated with DMSO or 2,4-PDCA were cultured with autologous CD4^+^ T cells in the presence of RSV to assess the APC function of the MoDCs (Fig 3D). While the T cells co-cultured with 2,4-PDCA-treated DCs had similar levels of IFN-γ production, the Th2 cytokines IL-5 and IL-13 were significantly decreased. These data suggest that inhibiting KDM5B function in human MoDCs results in regulation of Th2 cytokine production, supporting the hypothesis that RSV drives an altered immune phenotype that relies on epigenetic regulation of DC.

### Transfer of RSV-infected *Kdm5b*-deficient DCs leads to a decreased pathogenic pulmonary RSV challenge

**Fig 4:**
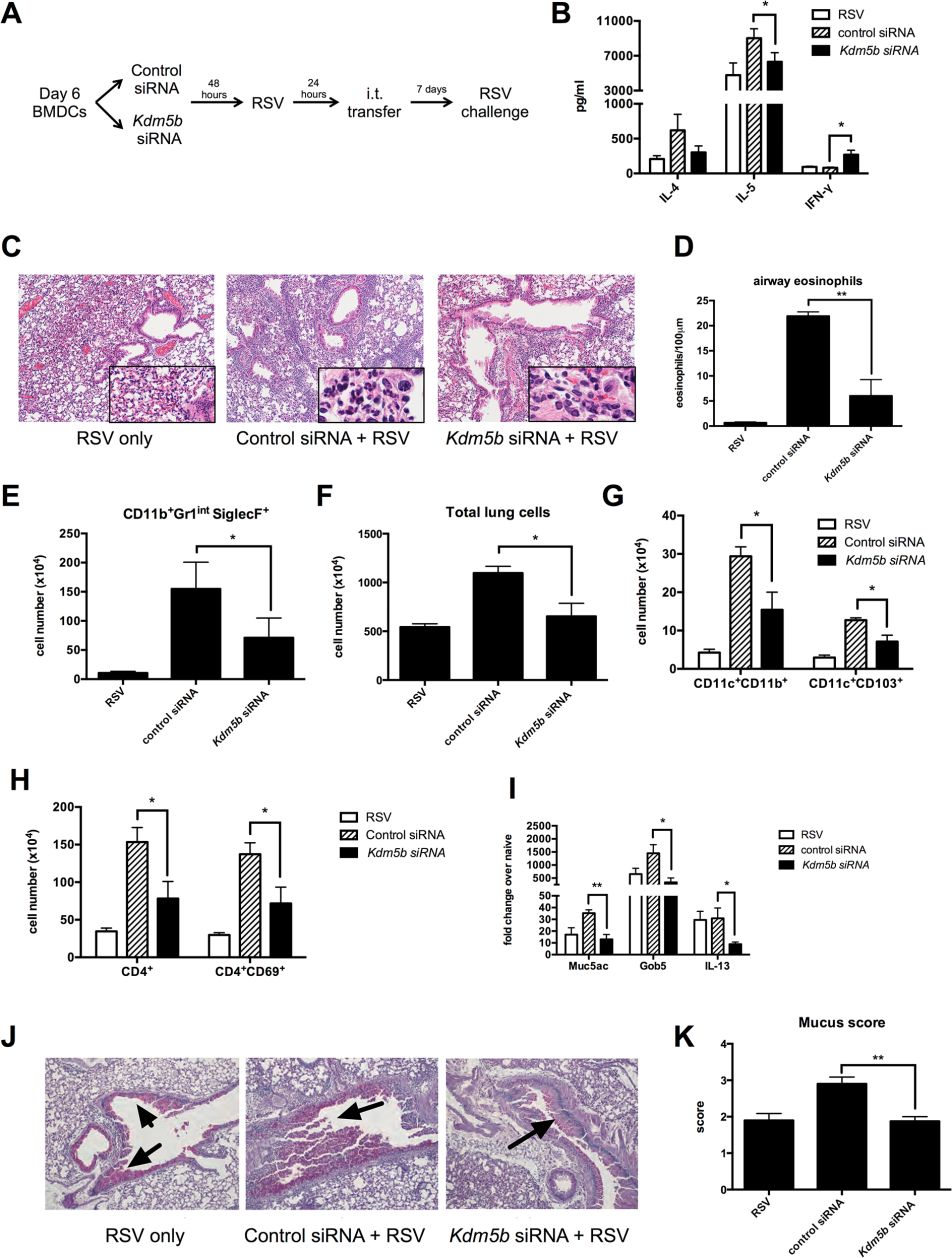
Transfer of RSV-infected *Kdm5b*-deficient DCs leads to a decreased pathogenic pulmonary RSV challenge. (A) BMDCs were transfected with *Kdm5b*-specific siRNA or non-targeting control, then infected with RSV overnight. These BMDCs were transferred intratracheally to the lungs of naïve mice 7 days prior to RSV infection. (B) At 8 days post infection, protein levels were measured from MLN cells restimulated with RSV *in vitro*. (C) Inflammation around the airways was measured by H&E staining in mice primed with BMDCs, including airway eosinophils (inset). (D) Eosinophils were counted in 100μm sections around the airways. (E) Lungs were dispersed into a single cell suspension using collagenase A and DNase. Esoinophils were measured using flow cytometry. (F) Total lung cells were counted following digestion of lung tissue. CD11c^+^CD11b^+^ and CD11c^+^CD103^+^ DC populations were measured by flow cytometry (G), as well as CD4^+^ T cells and activated CD4^+^CD69^+^ cells (H). (I) RNA was extracted from lung tissue using Trizol reagent, and transcripts of *Muc5ac*, *Gob5* and *Il13* were measured by qPCR. (J) Mucus was visualized in the lungs using PAS staining. Arrows indicate mucus production. (K) Identifying numbers on histology slides were blinded and slides were scored on a scale of 1–4 for mucus production. For all data n = 4-5samples/group and data are representative of two independent experiments. *p<0.05, **p<0.01.

Pro-inflammatory cytokines, including IFN-β, are important factors in driving Th1 responses following viral infection *in vivo* [26]. The above data indicated siRNA knockdown of *Kdm5b* led to increased pro-inflammatory cytokines *in vitro*, suggesting that priming the immune system *in vivo* with these DCs would drive a stronger Th1 response to RSV. To test this hypothesis, BMDCs were treated with *Kdm5b*-specific siRNA or scrambled control siRNA for 48 hours and subsequently infected overnight with RSV. These cells were then transferred intratracheally into naïve C57Bl/6 mice to prime the immune system in the context of *Kdm5b*-deficient DCs. One week after transfer, mice were challenged with RSV (Fig 4A). Our lab has previously demonstrated that this sensitization protocol with myeloid DC elicits a pathogenic immune environment upon RSV reinfection, and that the RSV-infected DCs that are transferred begin migrating to the lymph nodes within 24 hours [27,28]. Eight days following challenge, the mediastinal lymph nodes (MLN) were removed and restimulated *ex vivo* to determine the effect that sensitizing mice with *Kdm5b*-knockdown DCs would have on the T cell response. The data indicated an increase in IFN-γ production from mice that had been sensitized with *Kdm5b*-specific siRNA treated DCs, accompanied by a significant decrease in IL-5 and a trend toward less IL-4 production (Fig 4B). Furthermore, it was found that the lungs of mice primed with DCs that had been transfected with *Kdm5b*-specific siRNA had decreased inflammatory infiltrates compared to mice primed with control siRNA-treated DCs, as observed by H&E stained sections (Fig 4C). High-power images revealed that the inflammation surrounding the airways of control RSV-infected DC-sensitized mice contained numerous eosinophils (Fig 4C, insets). The number of eosinophils surrounding the airways was enumerated by microscopy in 100μm sections demonstrating decreased numbers in the *Kdm5b* siRNA group (Fig 4D) and confirmed with flow cytometric analysis of Gr-1^+^ and Siglec-F^+^ cells (Fig 4E). Additionally, it was noted that the total number of cells in the lungs was increased in mice sensitized with control siRNA, but the lungs had fewer cells when the mice received *Kdm5b* siRNA (Fig 4F). Flow cytometric analysis showed that these differences in total lung cells were due to fewer numbers of both conventional CD11c^+^CD11b^+^ DCs, as well as CD11c^+^CD103^+^ DCs (Fig 4G) [29,30]. Furthermore, there was a decrease in total CD4^+^ T cells as well as activated CD4^+^CD69^+^ cells (Fig 4H). Increased numbers of total cells, as well as DCs, T cells and eosinophils were also observed in mice that where sensitized with uninfected DCs, but to a lesser degree. (S5 Fig). In these mice, the numbers of inflammatory cells were similar to the numbers of cells measured in mice that received *Kdm5b*-deficient DCs, but was lower than the number of cells noted with control-treated, RSV-infected DCs were used to prime mice prior to challenge. Thus, the inhibition of *Kdm5b* in RSV-infected DCs led to significant protection from immunopathology that often has been associated with immunization to RSV [8].

Mucus production is a common occurrence during RSV infection, and is linked to the Th2 response to the virus. When mice were primed with *Kdm5b*-specific siRNA transfected DCs prior to RSV challenge, lower levels of the mucus-associated genes *Muc5ac* and *Gob5* were observed in the lungs of mice primed with *Kdm5b*-specific siRNA (Fig 4I). In addition, a primary inducer of goblet cell metaplasia and mucus hypersecretion, *Il13*, was also reduced in mice primed by *Kdm5b* siRNA inhibited DC (Fig 4I). Furthermore, mucus levels in the airways decreased, as visualized by PAS staining (Fig 4J). Scoring of mucus production on a scale of 1–4 is quantified in Fig 4K and demonstrated a significant decrease in overall mucus production in the *Kdm5b*-inhibited DC transfer model. These results indicate that priming the immune response to RSV with DCs lacking *Kdm5b* results in a less pathogenic immune environment *in vivo*.

### Infection of Kdm5b^f/f^-CD11c-Cre^+^ mice results in decreased Th2 cytokines and decreased mucus production following RSV infection

**Fig 5:**
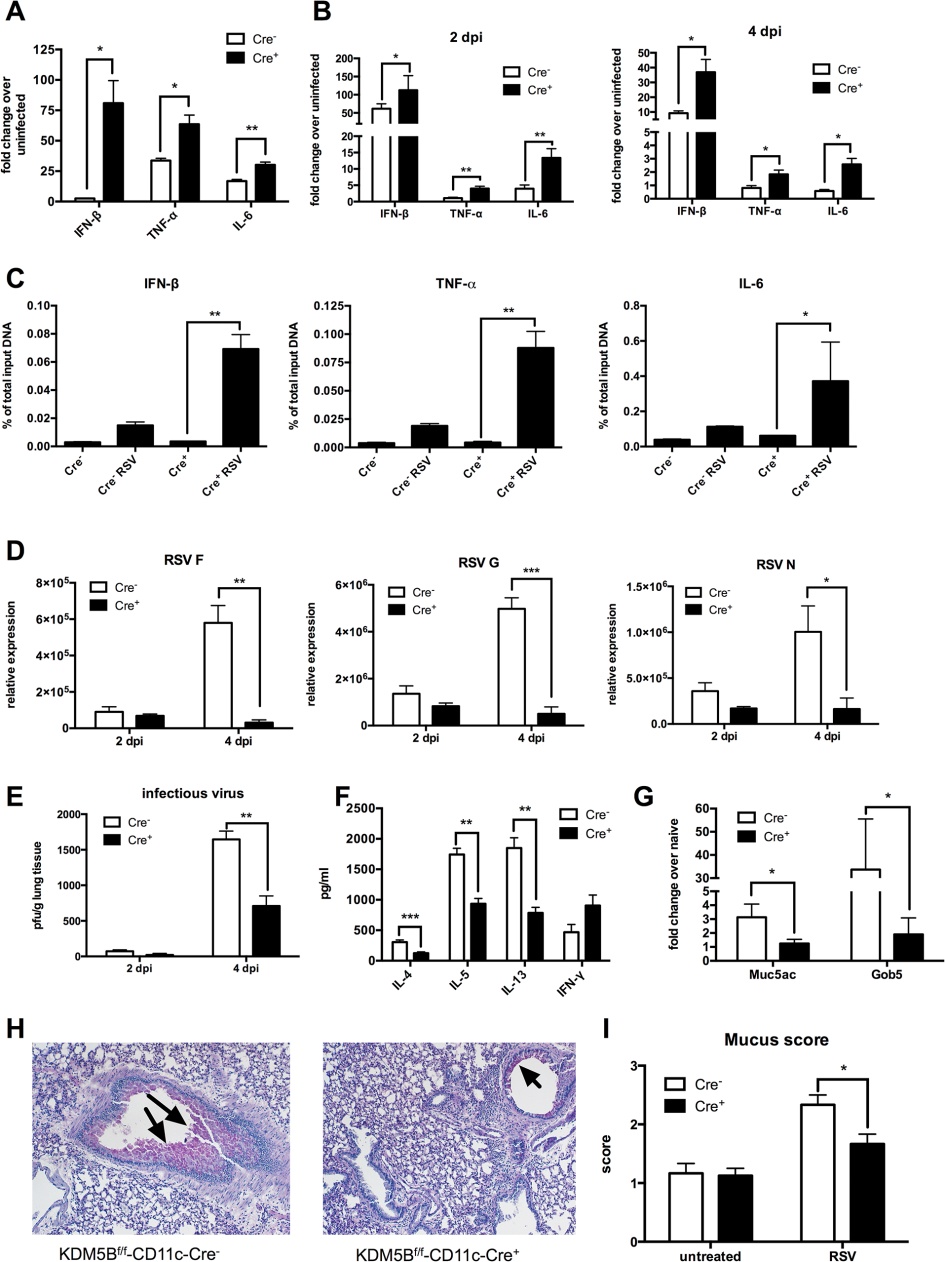
Deletion of *Kdm5b* in DCs leads to increased cytokine production and decreased Th2 pathology *in vivo*. (A) DCs were cultured from bone marrow of *Kdm5b* ^f/f^-CD11c-Cre^+^ mice and Cre^-^ controls and were infected with RSV. RNA was extracted after 24 hours and cytokine production measured by qPCR. (B) *Kdm5b* ^f/f^-CD11c-Cre^+^ mice and Cre^-^ controls were infected intranasally with RSV and the lungs were removed at 2 and 4 dpi. Lung tissue was homogenized and RNA extracted. Cytokine levels were measured by qPCR, and are expressed as fold change over infected Cre^-^ controls. (C) BMDCs were infected with RSV for 24 hours, and a ChIP assay was performed to determine the H3K4me3 status at the promoter regions of the *Ifnb*, *Tnf* and *Il6* genes. (D) Lungs were removed from mice at 2 and 4 dpi and viral mRNA levels for the F, G and N genes were measured by qPCR. (E) Infectious virus was determined by plaque assay at 2 and 4 dpi. (F) MLN were removed from mice at 8 dpi and were restimulated *in vitro* with RSV for 48 hours. Proteins in the supernatant were measured by bioplex assay. (G) Mice were infected with RSV for eight days, then lungs were removed and homogenized, and RNA was extracted. Transcripts of *Muc5ac* and *Gob5* were determined by qPCR. (H) Lung sections were stained with PAS to visualize mucus production at 8 dpi. Arrows indicate mucus production. (I) Mucus production was quantified from in lung sections on a scale of 1–4. *p<0.05, **p<0.01.

To provide further genetic evidence for the role of *Kdm5b*, a DC-specific Kdm5b knockout mouse was developed. *Kdm5b* ^f/f^ mice [31] were crossed with CD11c-Cre^+/-^ mice to create Kdm5b knockout CD11c^+^ DCs. Initial evaluation of these mice demonstrate no baseline differences in the number of immune cells in the lungs and spleens, including CD3^+^CD4^+^ and CD3^+^CD8^+^ T cells, CD11c^+^CD11b^+^ DCs and CD11b^+^F4/80^+^ macrophages (S6 Fig). BMDCs from *Kdm5b* ^f/f^-CD11c-Cre^+^ and control *Kdm5b* ^f/f^-CD11c-Cre^-^ mice that were grown had no identifiable growth or differentiation defects. BMDC from *Kdm5b* ^f/f^-CD11c-Cre^+^ mice infected with RSV demonstrated increased expression levels of the innate cytokines *Ifnb*, *Tnf* and *Il6* at 24 hours compared to Cre^-^ controls (Fig 5A), suggesting that KDM5B acts to suppress cytokine expression in DCs following RSV infection. These latter differences were not due to infectivity as there were no differences in the ability to infect the DCs from the Cre^+^ mice (S7 Fig). These mice were then infected with RSV, and cytokine transcripts were measured in whole lung tissue early after infection. Innate cytokine transcripts were elevated in Cre^+^ mice over Cre^-^ controls at both 2 and 4 days post-infection (dpi) (Fig 5B). To determine whether this corresponded to the demethylase activity of KDM5B at the promoter regions of these genes, a ChIP assay in *Kdm5b*-deficient BMDCs and Cre^-^ controls probing for H3K4 methylation was performed. Similar to observations using chemical inhibition of KDM5B, the *Kdm5b*-deficient DC had increased H3K4me3 at the promoter regions of all three inflammatory genes examined (Fig 5C) as assessed by ChIP analyses, but not of *Il10* and *Il12* (S4 Fig). These results further support the notion that KDM5B demethylates H3K4 at specific innate cytokine promoters, thereby contributing to gene suppression.

The previous data above suggested a decreased Th2 response to RSV infection when mice were sensitized with *Kdm5b*-knockdown DCs. However, to determine whether KDM5B is important in primary RSV infection, *Kdm5b* ^f/f^-CD11c-Cre^+^ and control mice were infected with RSV. At 2 and 4 dpi, lungs were removed and RNA was isolated from homogenates. mRNA levels of the RSV proteins F, G and N were measured (Fig 5D). Previous studies indicate that RSV replication peaks at 4 dpi [32]; no differences in RSV gene expression at 2 dpi were found, but by day 4, viral gene expression was significantly increased in *Kdm5b* ^f/f^-CD11c-Cre^-^ mice, whereas viral clearance was enhanced in *Kdm5b* ^f/f^-CD11c-Cre^+^ mice. These results were consistent with a plaque assay, where there were no differences in the levels of infectious virus at 2 dpi, but at 4 dpi the *Kdm5b* ^f/f^-CD11c-Cre^+^ mice had significantly fewer infectious particles in the lungs compared to controls (Fig 5E). At 8 dpi, MLN were removed and restimulated with RSV *ex vivo*. Similar to results shown in Fig 4, the data indicated that Th2 cytokines IL-4, IL-5 and IL-13 were substantially decreased in supernatants from restimulated cells, while IFN-γ production was increased, although not significantly (Fig 5F). Expression of the mucus-associated genes *Muc5ac* and *Gob5* in the lung tissue of infected mice was also measured. *Kdm5b* ^f/f^-CD11c-Cre^+^ mice had lower expression of mucus-associated genes compared to the control Cre^-^ mice (Fig 5G). Visualization of mucus by histologic PAS staining demonstrated decreased mucus production in *Kdm5b* ^f/f^-CD11c-Cre^+^ mice compared to controls (Fig 5H). Slides were scored on a scale of 1–4 for mucus production, and quantification showed less mucus production in *Kdm5b* ^f/f^-CD11c-Cre^+^ mice (Fig 5I). Together, these data demonstrate that genetic deletion of *Kdm5b* from CD11c^+^ cells results in increased innate cytokine production by DCs and a correlative decrease in the Th2 response to RSV in *vivo*.

### Sensitization with *Kdm5b* ^f/f^-CD11c-Cre^+^ BMDCs prior to RSV infection leads to decreased inflammation, Th2 cytokine expression and mucus production *in vivo*

**Fig 6:**
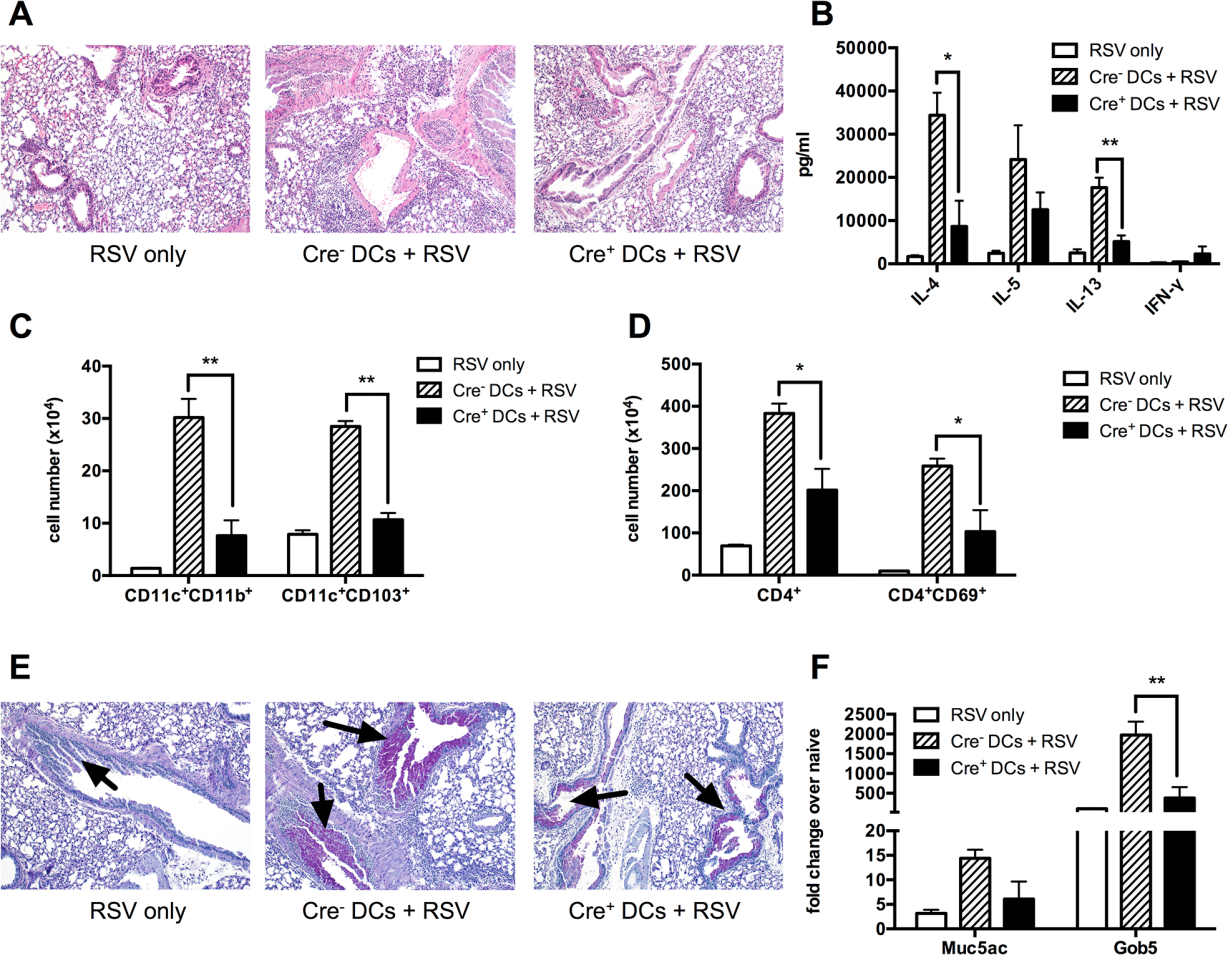
Sensitizing the lungs with *Kdm5b* ^f/f^-CD11c-Cre^+^ DCs prior to infection results in decreased pathology following RSV challenge. BMDCs from *Kdm5b* ^f/f^-CD11c-Cre^+^ mice or Cre^-^ controls were infected with RSV for 24 hours *in vitro*, then washed and administered intratracheally to WT C57BL/6 mice. After 7 days, mice were then infected with RSV, and samples were measured at 8 dpi. Mice infected with RSV alone were included as a control. (A) Lungs were removed and sections stained with H&E to visualize inflammation. (B) MLN were removed and restimulated *in vitro* for 48 hours with RSV. Supernatant proteins were measured by bioplex assay. (C) Lungs were homogenized into a single cell suspension and the number of CD11c^+^CD11b^+^ and CD11c^+^CD103^+^ DCs was quantified by flow cytometry. (D) CD4^+^ T cells and activated CD4^+^CD69^+^ T cells were measured by flow cytometry. (E) Lung sections were stained with PAS to visualize mucus production. Arrows indicate mucus production. (F) RNA was extracted from homogenized lung tissue and *Muc5ac* and *Gob5* were measured by qPCR. *p<0.05, **p<0.01.

The above studies found a decrease in proinflammatory cytokines in *Kdm5b* ^f/f^-CD11c-Cre^+^ BMDCs similar to that observed with siRNA-treated BMDCs. To confirm that our results in the *Kdm5b* ^f/f^-CD11c-Cre^+^ mice were mediated by DCs, BMDCs were infected with RSV for 24 hours, then delivered intratracheally into mice. After 7 days, mice were infected with RSV and assessed at 8 dpi. We found that mice that received *Kdm5b* ^f/f^-CD11c-Cre^-^ DCs had increased inflammation compared to RSV only controls, but that this inflammation was decreased in mice that were sensitized with *Kdm5b* ^f/f^-CD11c-Cre^+^ DCs, as determined by cellular infiltrates and H&E staining (Fig 6A). The MLN were removed and restimulated with RSV *in vitro* for 48 hours. Sensitizing mice with RSV-infected DCs led to increased levels of Th2 cytokines, IL-4, IL-5 and IL-13, but cytokine production was significantly lower when mice had been sensitized with *Kdm5b* ^/f^-CD11c-Cre^+^ DCs compared to controls, although the decrease in IL-5 was not significant. These mice also had an increase, although not significant, in IFN-γ production from the lymph nodes (Fig 6B). *Kdm5b* ^f/f^-CD11c-Cre^+^ DCs sensitized mice had fewer numbers of both CD11c^+^CD11b^+^ and CD11c^+^CD103^+^ DCs as well as fewer CD4^+^ T cells in the lungs. Also, fewer activated CD4^+^CD69^+^ T cells were observed in the lungs of Cre+ sensitized mice compared to mice sensitized with control DCs (Fig 6C and 6D). Finally, *Kdm5b* ^f/f^-CD11c-Cre^+^ DC sensitized mice had less mucus production in the lungs compared to mice treated with control DCs, with a significant decrease in *Gob5* expression (Fig 6E and 6F). Together, these results confirm the role of Kdm5b in skewing the immune environment towards increased pulmonary pathology.

## Discussion

**Fig 7:**
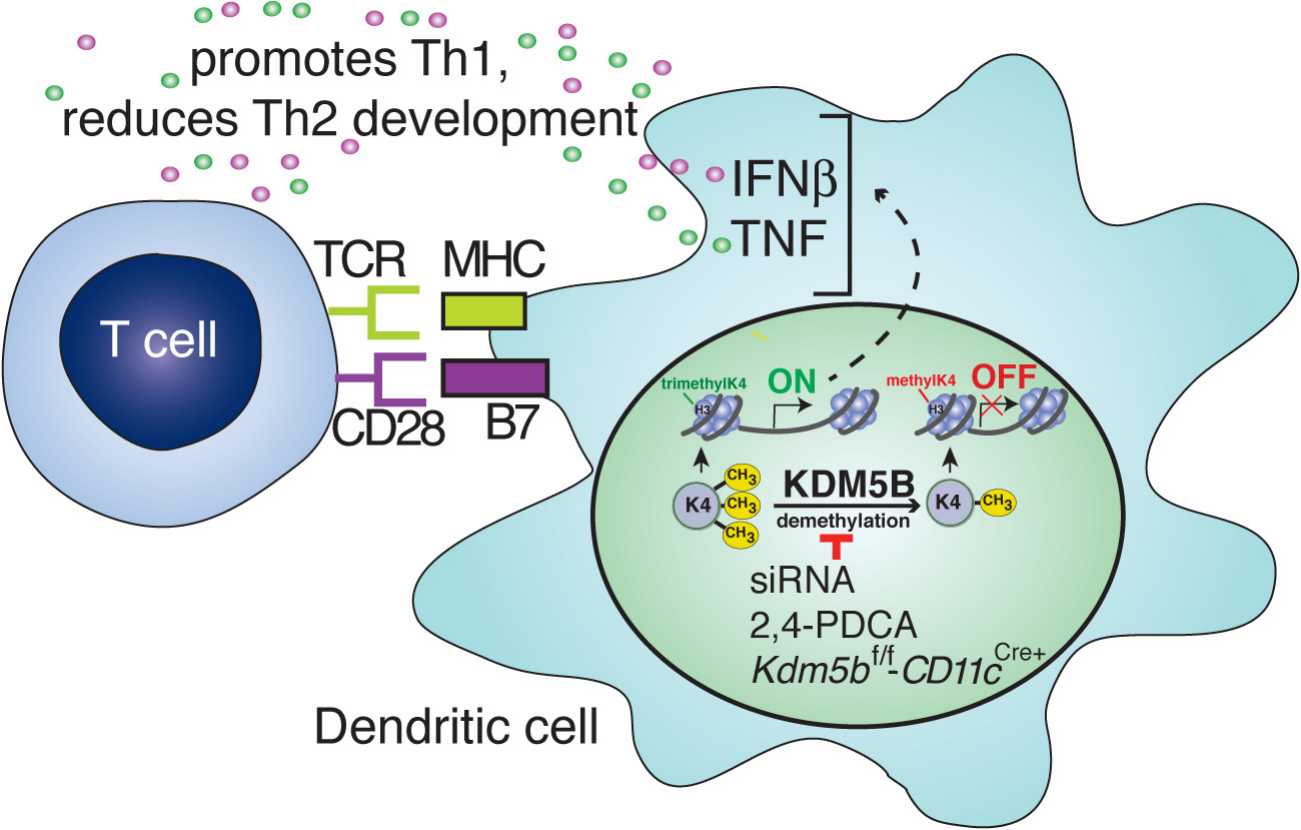
Blocking KDM5B demethylation leads to increased cytokine production and repression of Th2 responses. Following RSV infection, KDM5B is upregulated through unknown mechanisms. KDM5B demethylates H3K4 at the promoter regions of proinflammatory cytokines, leading to decreased transcription. Blocking *Kdm5b* by genetic means or siRNA silencing, or blocking KDM5B protein activity by chemical inhibition, leads to loss of demethylase activity, resulting in enhanced cytokine production from the DCs. Independent of MHC-II and costimulatory molecule surface expression on DCs, this increase in proinflammatory cytokines promotes Th1 responses and represses Th2 responses from the communicating T cells.

The objective of this study was to explore whether epigenetics are involved in determining the immune response during RSV infection. Previous studies have identified that DCs are a primary innate cell altered during RSV-induced pathogenesis, and that these cells play a central role in determining the nature of the immune responses. The concept that our immune system responses are influenced by environmental factors including microorganisms, pollution, diet and pathogen exposure is important as a backdrop for understanding disease progression [33–35]. Thus, we hypothesized that RSV infection of DCs would lead to the differential expression of epigenetic enzymes in these cells. The studies found that KDM5B, an H3K4 demethylase, was upregulated in DCs following RSV infection, but not when the cells were treated with ligands for TLR3 or TLR7, which are known to be important in recognition of the virus [22,23,36]. As H3K4me3 is an activation mark, it was further hypothesized that inhibiting KDM5B, which would remove methyl groups and thus remove the activation mark, would alter the activation state of the DC. Indeed, proinflammatory cytokines expressed by DCs were increased when KDM5B was blocked, which *in vivo* led to a suppression of the Th2 phenotype often associated with RSV infection. Thus, the present study identified several important concepts; 1) Epigenetic enzymes are a part of the mechanism by which a pathogen can modify the immune response; 2) Specific epigenetic enzymes have distinct effects on the function of DCs; 3) By identifying and specifically blocking epigenetic enzyme function a profound effect can be observed on the pulmonary immune environment. These results are summarized in Fig 7.

Changes in the lung environment due to pathogen exposure have been well documented. Viral infections often induce long-term alterations in both immune function and lung physiology. For example, infection with influenza leads to a subsequent increase in susceptibility to bacterial infections, which is mediated in part by excessive IL-10 production in the lungs [37] and increased neutrophil apoptosis [38]. Conversely, influenza infection has been shown to be protective of the Th2 response associated with subsequent RSV infection in a mouse model [39]. Similarly, RSV infection also induces long-term changes in the lung environment, particularly when severe infection occurs early in life. Numerous studies have linked infection in infancy with childhood wheezing and the development of asthma [5,6]. While the mechanisms of this connection remain unclear, the cytokine environment that develops during infection may contribute to the pathology. In severe RSV infections during early childhood, the immune response in children requiring hospitalization is often characterized by the presence of Th2 cytokines and associated with increased eosinophil infiltration and mucus overproduction [40,41]. During early life, the immune system is predominantly biased toward a Th2 phenotype, and a Th1 balance does not develop until about one year of age [42], which may contribute to the altered immune response to RSV infection in the lungs of infants. The data presented in these studies highlight a potential Th2 reinforcing response that is induced by RSV in DC that would limit the ability of an individual to develop a more appropriate, less pathogenic anti-viral response. This specific epigenetic mechanism, elicited by KDM5B, may be but one contributing factor that influences RSV pathogenesis.

RSV is relatively poor at inducing type I IFNs, especially IFN-β, compared to other viruses including influenza [9,43–47]. The production of IFN-β from DCs has been shown to be important in directing DC maturation and cytokine production, indicating that increasing IFN-β production by DCs could alter the immune environment following RSV infection [48]. Previous studies have highlighted the importance of IFN-β production in developing an antigen-specific response to RSV, and have found that STAT1-deficient mice, which cannot initiate signaling from either type I or type II IFN, have significantly increased Th2 responses, as well as increased illness [26,49]. On the other hand, IFN-γ-deficient mice are protected, indicating that type I IFNs are important for developing an appropriate anti-viral response to RSV [26]. Furthermore, IFN-β and TNF-α are important in establishing a Th1 response in humans, thus increasing levels of these cytokines could help skew an effective immune response to RSV [50,51]. In support of these previous findings, the present study demonstrates that a primary consequence of removing KDMB from DC is the increase in both IFN-β and TNF-α, resulting in reduced Th2 pathology. Alternatively, increasing proinflammatory cytokines from DCs that lack functional KDM5B protein resulted in increased viral clearance from the lungs following primary RSV infection. The resulting decreased antigen levels in the lungs may potentially lead to DCs being able to preferentially drive a Th1 phenotype over Th2. However, as there were no differences in the antigen presenting ability of DCs deficient in *Kdm5b*, it is likely that the cytokines produced from the DCs are the primary factor that leads to decreased Th2 pathology.

Dendritic cells are the primary cell type responsible for stimulating CD4^+^ T cells and directing the adaptive immune response. Many of the long-term immunologic changes that occur in the lungs following RSV infection are due to alterations in DC function [27,28,46]. Although RSV nonstructural proteins (NS1 and NS2) have been specifically implicated in the inhibition of type I IFN production [9,52–57], the molecular mechanisms have not been fully explored. Recent studies in epigenetic mechanisms have led to a greater understanding of the molecular control of immune cells in the context of infectious agents. It has been shown that long-term changes in DC function and cytokine production following an inflammatory insult are due in part to epigenetic changes in the *Il12* gene [17]. Others have demonstrated that the ability of DCs to prime Th1 responses is due to histone deacetylase activity [58]. More specifically, epigenetic enzymes can regulate the antiviral response by controlling the interferon pathway, especially IFN-stimulated genes [59]. This modulated immune effect was shown to be mediated by the G9a/GLP enzymatic complex [59]. In our studies, targeted gene arrays identified epigenetic enzymes that may contribute to the altered DC response following RSV infection. By using this unbiased approach, we targeted KDM5B as an important modulator of DC innate cytokine responses, as increased *Kdm5b* expression correlated with decreased *Ifnb* expression. By specifically inhibiting KDM5B and by generating a novel CD11c (DC) targeted KO mouse, studies identified that KDM5B has a critical role for altering DC function. Even more striking was the fact that the single enzyme alteration had a pathogenic impact on the anti-RSV response, including modifying T cell cytokine profiles and development of goblet cell metaplasia and mucus hypersecretion.

In summary, our studies identify a unique mechanism by which RSV infection influences how DCs are activated and subsequently activate the T cell responses leading to increased pulmonary pathogenesis. These studies used three methods to inhibit the expression or function of *Kdm5b*-siRNA knockdown, chemical inhibition and genetic deletion–and in all cases increased levels of innate cytokine production in DCs that subsequently led to a decrease in the Th2 response was observed. While previous attempts to create vaccines have led to increased Th2 pathology, these studies argue that future vaccine designs may need to consider the epigenetic programs that control pro-inflammatory cytokine production by DCs related to a protective immune response.

## Materials and Methods

### Ethics statement

All experiments on animal were performed in compliance with the guidelines of the Office of Laboratory Animal Welfare from the National Institutes of Health. Procedures were approved by the University Committee on the Use and Care of Animals at the University of Michigan (protocol PRO00004817, exp. 04/03/2016). All protocols using human cells were approved by the University of Michigan Institutional Review Board. For all experiments using human subjects, written informed consent was obtained from all donors.

### Mice and infections

C57BL/6 mice were purchased from Jackson Laboratory (Bar Harbor, ME, USA). *Kdm5b* ^fl/fl^ mice were previously described [31,60], and were crossed with B6.Cg-Tg(Itgax-cre)1-1Reiz/J (CD11c-Cre) mice (Jackson Laboratory). Mice were infected intratracheally with 1 x 10^5^ pfu of RSV strain A2001/2-20, a clinical isolate originally from Vanderbilt University, was propagated as described [32]. Viral stocks were grown in Hep-2 cells and concentrations determined by plaque assay. In some experiments, cultured dendritic cells were infected with RSV for 24 hours, then thoroughly washed and 2.5 x 10^5^ cells were transferred intratracheally into naïve mice.

### Plaque assay

Whole lungs were harvested at two and four days post infection and were ground with a mortar and pestle. Supernatants were diluted and incubated with Vero cells for four days. RSV plaques were detected using a specific polyclonal antibody (Millipore, Billerica, MA).

### Bone marrow-derived dendritic cell culture

Bone marrow was collected by flushing the femur and tibia of hind legs with PBS + 1% fetal calf serum (FCS). BMDCs were grown in RPMI 1640 supplemented with 10% FCS, L-glutamine, penicillin/streptomycin, non-essential amino acids, sodium pyruvate, 2-mercaptoethanol (ME) and 10 ng/ml of recombinant murine granulocyte macrophage-colony stimulating factor (GM-CSF; R&D Systems, Minneapolis, MN, USA). Cells were fed on days 3 and 5 with fresh GM-CSF. On day 6, cells were cultured with RSV (MOI = 1), 20 μg/ml of poly(I:C) or 1 μg/ml of imiquimod. For some experiments, cells were first treated for 24 hours with 1 mM 2,4-PDCA (Sigma, St. Louis, MO, USA), a chemical inhibitor of KDM5B [24,25], or with DMSO (0.1%) as a control, then were infected with RSV.

### Monocyte-derived dendritic cell culture and co-culture with CD4^+^ T cells

Fifty to 70 ml of blood was taken by venous puncture. Peripheral blood mononuclear cells were isolated from heparinized blood by Ficoll-Paque (GE Healthcare) purification. Briefly, blood was diluted 1:1 with sodium chloride and tubes were centrifuged at 400g for 30 minutes with the brake off. Cells were harvested from the interface of the Ficoll layer, and were washed and enumerated. Monocytes were isolated using anti-CD14 microbeads, according to the manufacturer’s instruction (Miltenyi Biotec, San Diego, CA, USA). Cells were cultured in RPMI 1640 with 10% human serum and L-glutamine, pen/strep, non-essential amino acids, sodium pyruvate, 2-ME, 40 ng/ml of recombinant human IL-4 and 40 ng/ml of recombinant human GM-CSF (both from R&D Systems). Cytokines were replenished on days 3 and 6, and cells were used on day 7. For some experiments, CD4^+^ T cells were isolated from the CD14^-^ fraction using the T cell isolation II kit (Miltenyi Biotec). Cells were then cultured with MoDCs in the presence or absence or RSV. At 48 hours, RNA was extracted and message levels of IFN-γ, IL-5 and IL-13 were determined by qPCR.

### Transfection of dendritic cells with siRNA

BMDCs were transfected with siRNA at day 6 of culture. 1 μM of ON-TARGETplus or scrambled control siRNA (Dharmacon, Pittsburgh, PA, USA) was transfected using the Amaxa DC nucloefection kit and an Amaxa Nucleofector (Lonza Inc, Cologne, Germany). Cells were then cultured for 48 hours in complete medium and knock-down was assessed by qPCR. Cells were then infected with RSV for 24 hours.

### RNA isolation and qPCR

RNA was extracted using TRIzol reagent (Invitrogen, Carlsbad, CA, USA) or RNeasy Mini Kit followed by the Cleanup Kit (Qiagen, Germantown, MD, USA) and following the manufacturers instructions. RNA isolated from tissues was first homogenized. Complementary DNA was synthesized using murine leukemia virus reverse transcriptase (Applied Biosystems, Foster City, CA, USA) and incubated at 37°C for one hour, followed by 95°C for 5 minutes to stop the reaction. Real-time quantitative PCR was multiplexed using Taqman primers with a FAM-conjugated probe and GAPDH with a VIC-conjugated probe (Applied Biosystems) to measure transcription of *Il6*, *Tnf*, *Il13*, *Il4*, *Ifng* and *Kdm5b*. Fold change was quantified using the 2^-ΔΔCT^ method. Custom primers were designed to measure *Ifnb*, *Muc5ac*, *Gob5* and RSV F, G and N RNA levels. All reactions were run on an ABI Prism 7500 Sequence Detection System or ViiA 7 Real Time PCR System (both from Applied Biosystems).

### Histopathology

Lungs were removed at 8 days post infection. The large left lobe of each lung was inflated by injection with 4% formaldehyde. Lungs were embedded in paraffin, and 5 μm sections were sectioned and stained with hematoxylin and eosin (H&E) to visualize inflammatory cells or with periodic acid-Schiff stain (PAS) to visualize mucus production. To score mucus production, slides were evaluated by a blinded observer. Sections were scored based on the following scale: 1 –minimal, 2—slight, 3 –moderate, 4 –severe.

### Flow cytometry

Lungs and mediastinal lymph nodes were removed and single cells were isolated by enzymatic digestion with 1 mg/ml collagenase A (Roche, Indianapolis, IN, USA) and 20 U/ml DNaseI (Sigma). Cells were resuspended in PBS with 1% FCS and Fc receptors were blocked with purified anti-CD16/32 (clone 93; BioLegend, San Diego, CA, USA). Surface markers were identified using antibodies (clones) against the following antigens, all from BioLegend, unless otherwise specified: CD11c (N418), CD11b (M1/70), CD103 (2E7), CD3 (145-2C11), CD4 (RM4-5), CD69 (H1.2F3), I-A/I-E (M5/114.15.2), CD80 (16-10A1), CD86 (GL-1), Gr-1 (RB6-8C5; eBiosciences, San Diego, CA, USA) and SiglecF (E50-2440; BD Biosciences, San Jose, CA, USA).

### Cytokine production from lymph nodes

Mediatstinal lymph nodes were removed and single cells were isolated by enzymatic digestion. 5 x 10^5^ cells were plated in 200 μl of complete medium and were restimulated with RSV for 48 hours. Supernatants were collected and levels of the cytokines IL-4, IL-5, IL-13 and IFN-γ were measured by Bioplex assay (Bio-Rad).

### Chromatin immunoprecipitation

Chromatin immunoprecipitation (ChIP) was performed using an assay kit (Millipore) with minor modifications. Briefly, cells were fixed in 1% formaldehyde, then lysed in SDS buffer. Cells were then sonicated using a Branson Digital Sonifier 450 (VWR, West Chester, PA, USA) to create 200–1000 bp fragments. The lysate was clarified by centrifugation, and 5% of the supernatant was saved to measure the input DNA. The remaining chromatin was incubated with 1 μg of anti-H3K4me3 antibody (Abcam) or control IgG (Millipore) and incubated at 4°C with rotation overnight. Immune complexes were precipitated with salmon sperm DNA/protein A agarose beads. Crosslinking was reversed by incubation at 65°C and samples were treated with proteinase K. DNA was purified by phenol:chloroform:isoamyl alcohol separation and ethanol precipitation. Primers for the promoter regions of IFN-β, TNF-α and IL-6 were designed using Lasergene software and DNA was amplified by qPCR using SYBR Green buffer (Applied Biosystems).

### Statistical analysis

Results are expressed and mean ± SE. Statistical significance was measured first by one-way or two-way ANOVA as appropriate, followed by a Student Neuman Keuhl’s post-hoc *t* test. A *p* value of <0.05 was considered significant.

## Supporting Information

S1 Fig *Kdm5b* expression is not increased in epithelial cells or in response to influenza. Epithelial cells and alveolar macrophages were infected with RSV, and BMDCs were infected with influenza. RNA was extracted and *Kdm5b* expression was measured by qPCR.
(TIFF)

S2 Fig Influenza, but not RSV, drives high levels of IFN-β production from DCs. BMDCs were infected with RSV or influenza, and RNA was extracted at 4 and 24 hours. *Ifnb* expression was measured by qPCR.
(TIFF)

S3 Fig siRNA knockdown of *Kdm5b* in BMDCs does not affect DC differentiation and activation. DCs were transfected with *Kdm5b*-specific siRNA or a non-targeting control, then infected with RSV. DC differentiation was measure by flow cytometry, along with the activation markers MHC-II, CD80 and CD86.
(TIFF)

S4 Fig H3K4me3 status is unaffected on IL-10 and IL-12 following RSV infection. A ChIP assay was performed to measure the H3K4 methylation status of the promoter regions of *Il10* and *Il12* in mouse and human DCs treated with 2,4-PDCA, as well as in DCs from *Kdm5b* ^f/f^-CD11c-Cre^+^ mice and Cre^-^ controls treated with RSV. Data are representative of two to three independent experiments, and each sample was run in duplicate.
(TIFF)

S5 Fig Transfer of uninfected and RSV-infected DCs prior to RSV infection. Mice were sensitized with RSV-infected DCs or untreated DCs, then challenged with RSV. Control mice were either uninfected or only infected with RSV. Total cell counts in the lungs were measured, and populations of eosinophils and CD11b^+^ and CD103^+^ DCs, as well as activated T cells, were determined by flow cytometry.
(TIFF)

S6 Fig *Kdm5b* ^f/f^-CD11c-Cre^+^ mice do not have any baseline differences in T cells or antigen presenting cells. Cells were isolated from the lungs and spleens of naïve KDM5B^f/f^-CD11c-Cre^+^ mice and Cre^-^ controls. CD4^+^ and CD8^+^ T cells, as well as CD11c^+^CD11b^+^ DCs and CD11b^+^F4/80^+^ macrophages were measured by flow cytometry.
(TIFF)

S7 Fig Infection of BMDCs from *Kdm5b* ^f/f^-CD11c-Cre^+^ and *Kdm5b* ^f/f^-CD11c-Cre^-^ BMDCs with RSV is equivalent. BMDCs were infected with RSV (MOI = 1) and cells were harvested at various timepoints post-infection. RNA levels of RSV F, G and N were measured by qPCR.
(PDF)
