# Supplementary figures and images for: RSV-Induced H3K4 Demethylase KDM5B Leads to Regulation of Dendritic Cell-Derived Innate Cytokines and Exacerbates Pathogenesis In Vivo
Source: PLoS Pathog. 2015 Jun 17;11(6):e1004978. doi: 10.1371/journal.ppat.1004978 (PMC4470918; doi:10.1371/journal.ppat.1004978)

### RSV F

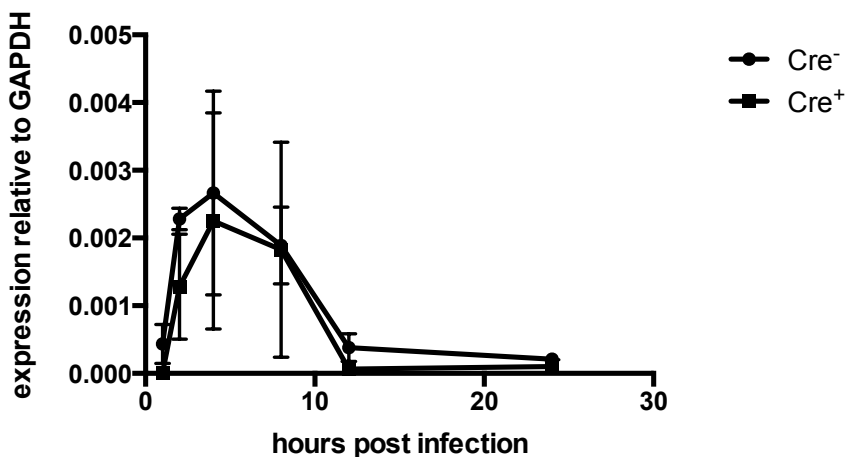

### RSV G

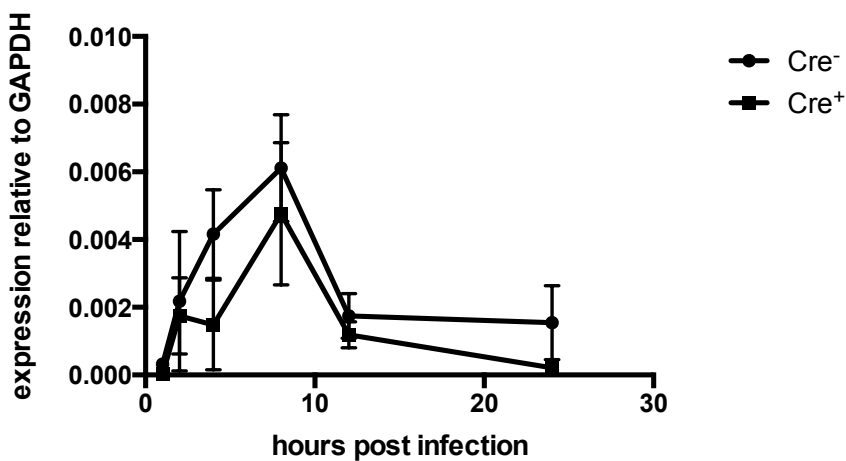

### RSV N

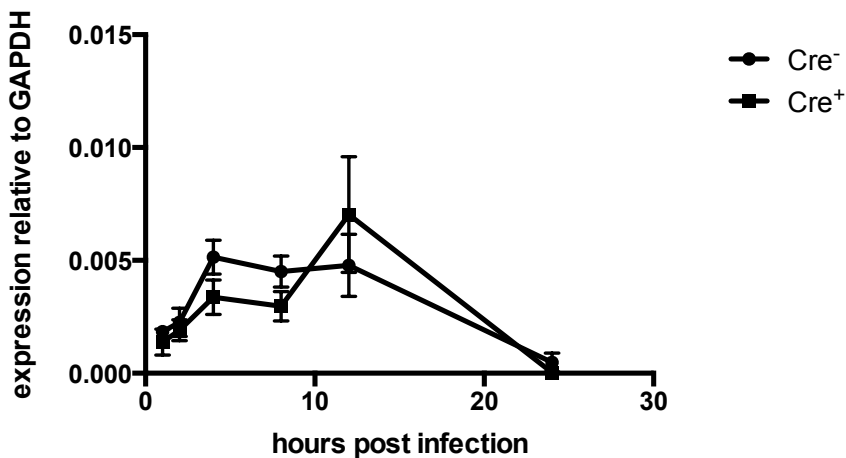

Supplement: S7 Fig — BMDCs were infected with RSV (MOI = 1) and cells were harvested at various timepoints post-infection. RNA levels of RSV F, G and N were measured by qPCR. (PDF) [file ppat.1004978.s007.pdf]
